# Surface Defects Control Bulk Carrier Densities in Polycrystalline Pb‐Halide Perovskites

**DOI:** 10.1002/adma.202407098

**Published:** 2024-10-31

**Authors:** David Cahen, Yevgeny Rakita, David A. Egger, Antoine Kahn

**Affiliations:** ^1^ Dept. of Mol. Chem. & Materials Science Weizmann Institute of Science Herzl 234 Rehovot 7610001 Israel; ^2^ Department of Materials Engineering Ben Gurion University of the Negev Be'er Sheva 8410501 Israel; ^3^ Department of Physics, School of Natural Sciences Technical University Munich James‐Franck‐Str. 1/1 85748 Garching Germany; ^4^ Department of Electrical and Computer Engineering Princeton University Princeton NJ 08544 USA

**Keywords:** defect tolerance, halide perovskites, self‐healing, surface defects

## Abstract

The (opto)electronic behavior of semiconductors depends on their (quasi‐)free electronic carrier densities. These are regulated by semiconductor doping, i.e., controlled “electronic contamination”. For metal halide perovskites (HaPs), the functional materials in several device types, which already challenge some of the understanding of semiconductor properties, this study shows that doping type, density and properties derived from these, are to a first approximation controlled via their surfaces. *This effect, relevant to all semiconductors*, and already found for some, is very evident for lead (Pb)‐HaPs because of their intrinsically low electrically active bulk and surface defect densities. *Volume* carrier densities for most polycrystalline Pb‐HaP films (<1 µm grain diameter) are below those resulting from even < 0.1% of surface sites being electrically active defects. This implies and is consistent with interfacial defects controlling HaP devices in multi‐layered structures with most of the action at the two HaP interfaces. Surface and interface passivation effects on bulk electrical properties are relevant to all semiconductors and are crucial for developing those used today. However, because bulk dopant introduction in HaPs at controlled ppm levels for electronic‐relevant carrier densities is so difficult, passivation effects are vastly more critical and dominate, to first approximation, their optoelectronic characteristics in devices.

## Graphical abstract

Doping, the controlled addition of imperfections or impurities to a material, is crucial for semiconductors. This study shows that for Halide Perovskites, which have (very) low (intrinsic) defect densities because of effective self‐healing and defect tolerance, the title's statement holds in an unprecedented fashion.

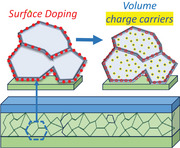

## 1 Introduction

We posit that electrically active defects at APbX_3_ lead halide perovskite (HaP) *surfaces* that form interfaces in devices or are present at interfaces in measurement and device configurations impact, and likely dominate, *bulk* electrical and electronic HaP properties and, ultimately, device performance. Mitigating the impact of these defects via passivation or other means remains therefore a major goal for controlling the behavior and improving the operational lifetime of HaP devices. At the same time, the very HaP properties that allow surface and interface defects to dominate electronic and electrical properties also enable doping of these materials via their surfaces and interfaces, a very important and potentially technologically relevant property. Before delving into the essence of this study, we recall some key concepts that are central to our arguments.

### 1.1 Semiconductor Surface

Semiconductor surface research over the past seven decades has led to a thorough understanding of how surfaces of inorganic and organic semiconductors affect electronic charge transport, as these surfaces ultimately form interfaces in devices. Yet, HaP surface and bulk behaviors challenge some aspects of this understanding. Here, we discuss and explain in which respects HaP surfaces that are part of interfaces with contacts or buffer layers differ from those of the nowadays well‐controlled (and understood) classical semiconductors such as Si, GaAs, CdTe, Cu(In,Ga)Se_2_ and, to some extent, the Pb‐chalcogenides.

### 1.2 Surface Defect States

Surface defect states (which can become interface states) can be intrinsic in nature, meaning that they result from the abrupt termination of the lattice (or lattice mismatch if occurring at an interface), or extrinsic, due to chemical changes that follow environmental conditions and/or impurities during processing (e.g., to form interfaces). In most inorganic semiconductors, the abrupt termination of the lattice results in dangling bonds corresponding to under‐coordinated atoms/ions at the surface. Such dangling bonds typically introduce electronic states with corresponding charge / discharge energy levels within the forbidden gap of the (bulk) semiconductor (E_G_). If these states are located around mid‐gap, or at least many times the thermal energy, *k*_B_ *T*, away from the conduction band minimum (CBM) or the valence band maximum (VBM), they are called deep levels. Defect states can be donor‐ or acceptor‐like, capture or release carriers, induce band bending and fix (“pin”) the Fermi level (E_F_) at and near the semiconductor surface. In classical covalently bonded semiconductors (e.g., Si), surface formation implies dangling bonds at the surface, intrinsic defects that induce near mid‐gap states. For some semiconductors, the surface atomic geometry can relax or reconstruct to minimize the surface energy, “sweeping” these dangling‐bond states away from mid‐gap. Surface relaxations can eventually lead to surface states resonant with the semiconductor CBM and/or VBM, making them (opto)electronically innocuous for the semiconductor. Typical examples are the non‐polar (110) surfaces of most III‐V compound semiconductors, whose dangling bond energies are close to, or overlap with CBM or VBM states.^[^ ^1^, ^2^, ^3^, ^4^, ^5^, ^6^ ^]^ We can further distinguish between “intrinsic” surface defects corresponding to atomic vacancies, anti‐site defects or interstitials, or “extrinsic” ones due to contamination by foreign species (often implied by temperature‐independent characteristics). At a surface, such defects (i.e., those beyond intrinsic ones due to the formation/existence of the surface itself) generally require a lower energy of formation than in the bulk.^[^ ^7^ ^]^ Like surface states, both intrinsic and extrinsic surface defects typically also induce donor‐ or acceptor‐like electronic levels within the semiconductor gap and can entail similar consequences for optoelectronic and device characteristics.

### 1.3 Inorganic Semiconductor Surface

Inorganic semiconductor surface formation means breaking ionic, covalent, or mixed ionic‐covalent bonds, leaving reactive, oxidizable/reduceable sites (including under‐coordinated species) at the surface. These phenomena are well‐known to occur in classical inorganic semiconductors such as the tetrahedrally bonded Si, III‐Vs (e.g., GaAs, InP), II‐VI (e.g., CdTe, CdSe), chalcopyrite (e.g., CuInSe_2_) and kesterite (e.g., Cu_2_SnZnS_4_) compounds, and somewhat less so for the octahedrally bonded IV‐VI materials (e.g., PbS, PbSe). Surface defect states of those inorganic semiconductors typically result in E_F_ pinning deep in (near the middle of) the gap,^[^ ^8^ ^]^ which can prevent the control of interface energy level alignment with adjacent layers, including metal‐semiconductor contacts. In a later section we consider cases where surface‐doping effects were found to be relevant for established semiconductors.

### 1.4 Organic Semiconductor Surfaces

Organic semiconductor surfaces rarely show dangling bond‐ or point defect‐like surface states, mainly because the basic molecular constituents are generally closed‐shell systems often held together by relatively weak interactions (e.g. van der Waals forces). One clear sign of this situation is the ability to move the Fermi level of an organic film over a sizable fraction of E_G_ by changing the work function of the substrate,^[^ ^9^, ^10^ ^]^ which is possible only in the absence of deep surface defect states. Instead, a charge exchange between organic film and substrate takes place, driven by differences in work function and unimpeded by any deep gap states at either the organic surface/interface or in the bulk. Such movement of the Fermi level throughout the gap of the organic semiconductor is also enabled by the very low intrinsic carrier density in organic films. However, Fermi‐level pinning states can be induced at organic surfaces if chemical reactions take place that open the closed‐shell structure of the molecules. Such situations have been reported, for example with (reactive) metals evaporated on organic surfaces,^[^ ^11^, ^12^, ^13^ ^]^ a process that is akin to the active formation of extrinsic states on inorganic semiconductor surfaces.

## 2 Halide Perovskites

### 2.1 Halide Perovskite Surfaces

**Figure 1:**
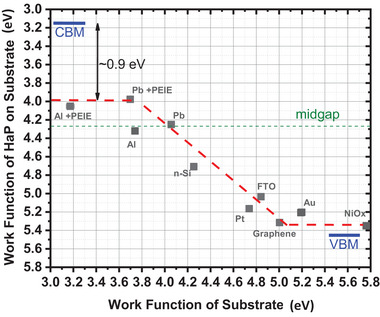
Plot of work function of a (FA_0.85_MA_0.1_Cs_0.05_)PbBr_3_ film deposited on different substrates, vs. substrate WF, showing the linear dependence, within a wide energy window, as noted in the text (reprinted with permission from ref. [14]; copyright 2019 American Chemical Society).

While various aspects of HaP surfaces have been and continue to be studied,^[^ ^15^ ^]^ the present analysis is strongly influenced by directions outlined in a previous review on HaP interfaces by two of the present authors.^[^ ^16^ ^]^ A major open question is whether HaP surfaces can be assigned to one of the semiconductor families mentioned above, i.e., inorganic, or organic, to a combination of the two, or if they should be considered as a third type with unique characteristics. We and others have found that, for films of several HaP types deposited on multiple substrates, E_F_ can move through much of E_G_ as a function of the substrate work function (**Figure** **1**).^[^ ^14^, ^17^, ^18^, ^19^, ^20^ ^]^ The data shown in Figure 1 were obtained in an inert atmosphere glove box. We note that these measurements and others from studies referred to above, give the positions of E_F_ in the gap and values of the work function *at the free HaP film surface*, i.e., several hundreds of nanometers away from the contact with the underlying substrate. However, the results show not only that the density of deep gap states at the free surface is very small, say ≤ 10^10^ cm^−2^ (≤ 10 ppm for surfaces of inorganics like c‐Si or BaTiO_3_), but also that the density of deep gap states at the buried (substrate/HaP) interface is comparatively low, and that the density of deep gap states in the *bulk* of the material is low as well, say ≤ 10^15^ cm^−3^ (cf. ref. [21] for values from devices). This triple characteristic enables the Fermi level to respond *throughout the material* to charge exchange as the material reaches (electronic) thermal equilibrium with the substrate.

**Figure 2:**
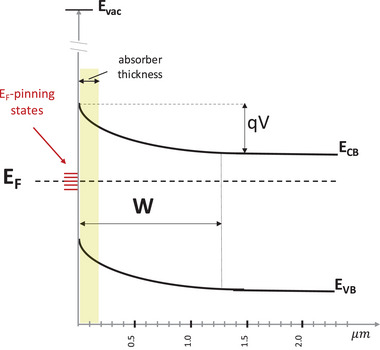
Schematic energy diagram of an n‐type semiconductor with deep gap states pinning the Fermi level, E_F_, at the surface/interface, and depletion region width, W, given by Equation 1. E_CB_ and E_VB_ are the conduction and valence band minimum and maximum, respectively. The highlighted region within the semiconductor, next to the surface, indicates what is relevant for W >> grain‐diameter; the energy band distance profile is then better described as band “slanting” instead of bending and there is no field‐free region in the grain (as there is in Figure 2, right hand side).

As shown in **Figure** **2**a, from a semiconductor surface inward there is typically a space charge layer, depleted of, or, in rare cases, with an accumulation of majority charge carriers. The space charge layer width, i.e., the depth *W* from the surface over which there is carrier depletion and thus band bending, is given by

(1) $$W=\sqrt{\frac{2\varepsilon_{s}V}{qN_{D}}}$$

where ɛ_*s*_ is the (static) permittivity of the semiconductor (halide perovskite here), *q* the electron charge, *N_D_* the dopant density and *V* is the voltage difference across the sample. Taking *V* = 0.1–1 V, ɛ_*s*_ ≈ 30 ɛ_*o*_ and *N_D_* = 10^13^ cm^−3^ (an upper limit for non‐contacted grains in films, based on measured dopant densities in single crystals) yields *W* ≈ 5–15 µm, i.e., many times the thickness/diameter of the film/grains (≈0.3–0.5 µm thick; the Debye length is ≈0.5 µm). Even for films in good quality HaP devices, i.e., with contacts, *N_D_* rarely exceed 10^16^ cm^−3^ (see explanatory note ‐A‐ below, just before the references), which still gives an upper limit of *W* ≈ 0.2–0.6 µm. Thus, E_F_ moves across the gap of an entire film. When *W* exceeds the grain‐thickness, one can consider that the entire film is depleted. In that case, charge exchange with the substrate can move E_F_ throughout the film *to its free surface*, i.e., even hundreds of nanometers away from direct contact with the substrate. As a result, the work function of the film becomes sensitive to the work‐function of the substrate on which the film is deposited, as illustrated in Figure 1.

Besides the wide space‐charge region, the energy landscape imposed by surfaces and interfaces in HaPs is shallow. Typical variations of the surface potential between grains in thin polycrystalline HaP films, as measured by scanning Kelvin probe force microscopy (SKPFM),^[^ ^22^, ^23^, ^24^ ^]^ are ≈15–40 meV,^[^ ^22^, ^23^ ^]^ quite small compared to what is found in as‐grown polycrystalline ‘classical’ semiconductors. Surface band bending into these grains is comparatively small.^[^ ^25^ ^]^ Such small band bending is consistent with a low volume doping and the absence of a significant density of deep surface/interface defect states. In contrast, for classical polycrystalline inorganic semiconductor films, surfaces and grain boundaries (GB), unless carefully passivated, present 100s of meV barriers for electronic transport.^[^ ^26^ ^]^

### 2.2 HaP Surface Composition and Defect States

APbX_3_ halide perovskite surfaces are terminated by A and X, or Pb and X species, where Pb and X are the more tightly bound elements (forming corner‐sharing octahedra, with A cations between them). The presence of any X or Pb species at the surface implies breaking one or more of the mixed ionic‐covalent Pb─X bonds. In view of the lessons learned from the behavior of organic semiconductor surfaces, it was originally thought that the presence of organic A cations at the surface did explain the apparent inertness of the Pb‐HaP surface and the relatively smooth surface energy landscape. Yet, the same (SKPFM)^[^ ^27^ ^]^ characteristics are observed at surfaces and interfaces of all‐inorganic Pb‐HaPs like CsPbBr_3_.^[^ ^24^ ^]^

Therefore, key questions pertain to the energy of electronic states due to (intrinsic) dangling bonds on HaP surfaces and the role of surface relaxation or reconstruction, if there is any. Furthermore, the presence of defect states at HaP surfaces and their energies, and their impact on the electrical and electronic properties of the bulk material, remains to be understood. Relevant in this context, recent work used nonadiabatic molecular dynamics to show that halide vacancies at CsPbBr_3_ surfaces surprisingly exhibit a reduced tendency to form deep defect states compared to their bulk counterparts.^[^ ^28^ ^]^ In line with this, the above‐ discussed experimental results clearly point to the absence of a significant density of deep gap states and to the ability to move E_F_ at surfaces that have not been significantly distorted by chemical reaction with extraneous species or by active defect formation via electron or other particle bombardment, by strong illumination or X‐ray beams.

### 2.3 HaP Surface Passivation

Pb‐HaP surfaces can be, and have been, successfully passivated. Impressive improvements in photoluminescence (PL),^[^ ^29^ ^]^ improved photovoltaic (PV) performance for polycrystalline film‐based devices,^[^ ^30^, ^31^ ^]^ as well as longer lifetimes of photogenerated carriers have all been reported.^[^ ^32^ ^]^ These findings suggest that surface defects are active in these materials, at least before such passivation occurs. Surface defect passivation is also important for single crystals of HaPs, as shown in experiments with single crystal‐based solar cells.^[^ ^21^, ^33^ ^]^ With the stage set, we can now address the subject given by the title of this study.

## 3 Volume and Surface Defect Densities and Energy Level Fluctuations

This section provides a feasibility argument for the surface doping hypothesis. Whether passivation eliminates some or all dangling bonds and defects at surfaces is unclear at this point. However, we recall that, in contrast to inorganic semiconductors, very respectable performances are already obtained with (3‐D) HaP‐based devices without any surface passivation.^[^ ^30^, ^31^, ^32^ ^]^ This implies that *bulk as well as surface defect densities can be very low compared to as‐prepared conventional inorganic semiconductors, already for non‐passivated HaPs*,^[^ ^34^, ^35^ ^]^ likely reasons for which will be discussed later.

### 3.1 The Simple Arithmetic of Bulk Doping by Surface Defects

**Figure 3:**
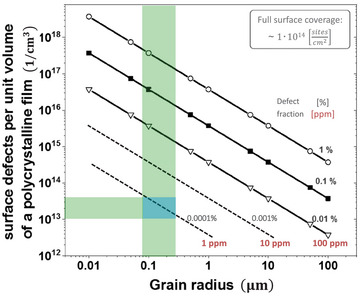
Equivalent volume defect density (Y‐axis) corresponding to 1 ppm to 1% of surface sites being defects, as a function of (spherical) grain radius (X‐axis). Volume defect density equals the number of surface defects divided by the volume of the bulk (cf. explanatory note ‐B‐). Typical range of grain radii in polycrystalline HaP films is shown by the vertical (green) column, and an example volume defect density (10^13^ cm^−3^) for 1 ppm defective surface sites is indicated by the horizontal bar (adapted from ref. [35]). Full surface coverage of sites for potentially electrically active defects is taken as indicated, which corresponds to roughly one site per unit cell exposed surface area of a typical Pb‐HaP. Similar figures can be found in the literature, e.g., in ref. [34].

**Figure 4:**
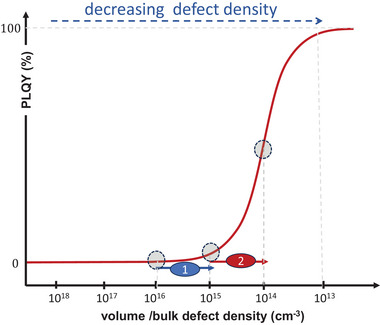
Schematic plot of change in photoluminescence quantum yield (PLQY) with changing density of defects that act as recombination and/or trapping centers. When the defect density is high, a reduction in density (arrow 1) yields a modest change in PLQY; when the defect density is low, the same order of magnitude reduction (arrow 2) leads to a significantly larger increase in PLQY. Note that the y‐axis is presented as a linear scale.

To understand the impressive PL increase upon surface passivation (from several‐fold to order of magnitude), we provide a step‐by‐step, quantitative calculation of how to translate the surface defect density (in cm^−2^) at grain surfaces and interfaces of a polycrystalline semiconductor film into an overall bulk defect density (in cm^−3^) in that film (see explanatory note ‐B‐); in refs. [7, 34], a similar geometric argument is given. Thus, we consider the *effective* bulk defect density to be the volume density of defects that equals the number of surface and interface defects divided by the volume of the bulk. **Figure** **3** shows that, for a common semiconductor film with ≈500 nm diameter spherical grains, low *effective* bulk defect densities, say, 10^15^ cm^−3^, can be achieved with < 0.01% (100 ppm) of the grains' surface sites being defective.^[^ ^1^ ^]^ Such density can depress the PL yield to a few %. However, the PL was found to rise spectacularly to 10s of % or even close to 100% upon further passivation of the surface to leave an equivalent of some 1–10 ppm defective sites, as illustrated in **Figure** **4** and reported experimentally (cf. e.g., ref. [36]).

The relation, shown in Figure 4, is not unique to Pb‐HaPs, but, due to their relatively low surface and bulk defect densities, is readily achieved with them. Ref. [37] gives an example for the organic molecular solid rubrene, and ref. [38] for monolayer MoS_2_. Using simulations of bulk defect density as function of grain size for given grain boundary/surface defect densities, ref. [34] shows how grain size can affect the photoluminescence quantum yield (PLQY). A drive level capacitance study on the distribution of trap states in space and energy of single crystal and polycrystalline Pb iodide perovskite‐based solar cells showed that trap state densities were low, and smaller by one to several orders of magnitude in the bulk than at surfaces/interfaces.^[^ ^21^ ^]^

Effects of crystallite size and/or orientation on PL emission intensity of MAPbI_3_ thin films were studied by Jariwala et al.^[^ ^39^ ^]^ In addition to a clear effect of orientation spread (higher PL intensity with narrower spread), some correlation between orientation and grain area was also found, but grain orientation was thought to dominate grain size. In relatively early work de Quilettes et al.^[^ ^36^ ^]^ already found, also using PL intensity as an indicator, higher PL emission intensity from the bulk away from grain boundaries, and an overall intensity increase after exposing the film to pyridine, used as surface passivating agent.

### 3.2 Relevant Computational Results

The fact that HaPs feature low energy anharmonic vibrations appears to be an important determining aspect for their defect characteristics. DFT computations that consider lattice dynamics via MD calculations showed^[^ ^40^ ^]^ that the (dis)charge electronic energy level of V_Br_ in bulk CsPbBr_3_ fluctuates strongly in energy (up to ± 0.5 eV) over a time scale of several ps, much longer than that found for an anti‐site defect in GaAs.^[^ ^40^ ^]^ At their extrema, these fluctuations can create significant overlap between the defect level and the states at the band edges. This mechanism could enable the transfer of charge to and from the band, thus allowing charging or discharging of these trap states, making them far less effective Fermi level pinning traps. This rationale can be understood considering a scenario where a trap state does not show significant thermally induced energy fluctuations: it will then not exhibit increased overlap with band‐edge states and increased likelihood of charging/discharging and remain a trap state for a longer time. Such behavior in this most ionic of the bromide perovskites is a vivid demonstration of the way the strong lattice dynamics in HaPs upset some of our long‐established concepts in semiconductor physics, in this case that of a well‐defined energy level for a given defect.

Subsequent DFT work found the effect (for V_I_) in MAPbI_3_ to be even stronger.^[^ ^41^ ^]^ Possible extrapolations of the lattice dynamics and associated defect level fluctuations from the bulk to the surface need further investigation, as highlighted in a few studies. Previous work by Ambrosio et al.^[^ ^43^ ^]^ found that trapping of holes preferably occurred at surfaces and suggested that passivation could prevent certain degradation mechanisms. Focusing on CsPbBr_3_ NCs, ten Brinck et al.^[^ ^42^ ^]^ assigned their “defect tolerance” to vacancies being difficult to form energetically at HaP surfaces. Further work will be required to elucidate in particular the fluctuations of energy levels of surface defects and their role in (dis)charging processes via temporal overlap with the CBM or VBM.^[^ ^40^, ^41^ ^]^ Interestingly, it has been reported that these level fluctuations are reduced at the surface compared to the bulk,^[^ ^28^ ^]^ yet the defect‐induced states may remain shallow enough and show sufficient overlap with band‐edge states to possibly promote defect tolerance.

### 3.3 Self‐Healing and Defect Tolerance

Figure 3 shows that if the defect density in polycrystalline HaP thin films is below ≈10^16^ cm^−3^, this corresponds to < 0.1 at.% of surface sites of sub‐µm grains being electrically active. The relatively low bulk defect densities in these materials have been explained by self‐healing and defect tolerance, which are both associated with lattice dynamics, as exemplified in the aforementioned computational studies.^[^ ^28^, ^40^, ^41^, ^42^, ^43^ ^]^ Also the dynamic chemical process of self‐healing of defects should encounter smaller barriers to movement of atoms/ions at the surface than in the bulk (as can be seen in melting of materials: surfaces melt at significantly lower temperatures than the bulk^[^ ^44^ ^]^), *as long as no material is lost or extrinsic material added*. If surface defects cannot self‐heal, their energy levels may still be tolerated if the energy level fluctuations are larger than their depth within the bandgap. Thus, both self‐healing and some defect tolerance can explain the apparent lack, or at least paucity, of deep E_F_‐pinning defect states at these surfaces. What cannot be healed is a defect due to loss of material from the surface, such as A or X vacancies, which then requires passivation by an external source, i.e., repair. This discussion illustrates the basis for control of volume electrical properties of HaP films by way of their surfaces.

Summarizing this part, passivation effects in/on HaP films can be highly effective for PL increase because densities of (opto)electronically active defects within film grains are intrinsically (very) low and can be modest to low on grain surfaces, already for as‐prepared films. These low defect densities make these materials more sensitive to smaller *changes* in active surface defect densities than other inorganic semiconductors. Leading evidence is the strong PL intensity rise upon surface passivation. This result points to the already low surface defect density of the material because, as noted above and illustrated schematically in Figure 4, an increase in semiconductor PL intensity with (surface) passivation is much more striking if an already low (surface defect) density is decreased further, as the PL yield increases exponentially with increasing passivation.

## 4 Is the Importance of Surface Defects for Bulk Carrier Densities Unique to HaPs?

### 4.1 History of Surface to Bulk Doping

The phenomenon was described already in 1955 when applying semiconductor theory to heterogeneous catalysis.^[^ ^45^ ^]^ Directly relevant to the present topic, the effect of oxygen on PbS doping was described as early as 1955.^[^ ^46^ ^]^ In the 1980s, surface oxidation (mostly oxygenation) effects, with little attention to doping, were studied also for Si and Ge.^[^ ^47^ ^]^ Around the same time the effect of oxygen on doping of CuInSe_2_ (in studies of solar cells; precursor to CIGS)^[^ ^48^, ^49^, ^50^ ^]^ was described, explained and generalized to binary chalcogenides, such as CdTe.^[^ ^51^ ^]^ DFT computations followed, for Pb chalcogenide nanoparticles^[^ ^52^ ^]^ and for CuInSe_2_ and CdTe^[^ ^53^, ^54^ ^]^ grain boundary surfaces and interfaces, as well as other compounds including Pb dihalides,^[^ ^55^ ^]^ diamond and SiC (where the phenomenon was re‐named *surface transfer doping*),^[^ ^56^, ^57^ ^]^ organic semiconductors^[^ ^56^, ^58^ ^]^ and transition metal dichalcogenides.^[^ ^59^ ^]^ Two very recent reports that are relevant are a review on grain boundaries in polycrystalline optoelectronic materials^[^ ^60^ ^]^ that compares HaPs with Pb‐chalcogenides, GaAs, CIGS and CdTe, and a very detailed experimental review on CuInSe_2_.^[^ ^61^ ^]^ Results consistent with surface doping, nowadays also called ionosorption, are important for sensors, such as SnO_2_,^[^ ^62^ ^]^ which were developed empirically in the early 1960s (Takuchi O_2_ detectors); their mode of action (see ref. [63] for a recent review) started to be elucidated in the mid‐to‐late1980s^[^ ^64^ ^]^ when this was done also for the chalcopyrite, CuInSe_2_ (ref. [48]).

This short survey shows that the HaPs are not unique in surface adsorption and desorption of various species leading to volume doping of bulk grains; the defect chemical description, given in explanatory note ‐C‐ for an exemplary HaP, can with minor modifications be applied to all the materials for which reversible surface sorption is possible and is itself adapted from such description for a chalcopyrite. Still, while not unique, the effect is particularly strong in HaPs because of the low intrinsic bulk and surface doping/defect densities present in this class of semiconductors.

### 4.2 Example of Surface Chemical Defect, Leading to Bulk Doping

A specific theoretical example of how a *surface* defect site can dope the HaP solid contained within the surfaces is given in explanatory note ‐C‐. For simplicity, we consider a well‐defined (static) point defect at the surface, i.e. the halide (X in the halide perovskite ABX_3_) surface vacancy, V_X_. Such a defect decreases the coordination of the neighboring Pb and leads to a Pb dangling bond. To illustrate the process, we use iodide as the constituent halide and chloride for surface iodide vacancy filling. This example thus helps to understand the basis for the hypothesis that *the volume electrical properties of polycrystalline HaP films can be modified by way of their surfaces*, rather than by the established way to control bulk doping of semiconductors, which requires first thorough purification of the bulk and passivation of their surfaces. While the analysis is a general one, it is particularly relevant for HaPs, because the success of standard bulk doping methods has been, at best, limited for this class of materials,^[^ ^65^ ^]^ a finding that is consistent with the ability of HaPs to heal defects (above the minimal defect density, dictated by thermodynamics).^[^ ^66^ ^]^ Doping limitations may also result from structural defects that are not optoelectronically active, that is the already discussed defect tolerance.^[^ ^67^, ^68^ ^]^ Generalizing, if self‐healing is effective, then control over electronic charge carrier type and densities relevant for doping (say, 10^14^–10^18^ cm^−3^) by methods such as ppm‐level valence substitution^[^ ^65^ ^]^ becomes problematic.

## 5 Survey of (More) Experimental Results Relevant to the Working Hypothesis

### 5.1 Overview of Reviews

The express objective of this summary is to challenge possible confirmation bias in support of the hypothesis. To that end, we consider first recent reviews relevant to the topic. Bao and Gao^[^ ^69^ ^]^ in a survey of defects physics in HaPs stress the shallow nature of the (computed) surface and grain boundary defect states, with low recombination activity, while deep traps of bulk plus interface defects are noted. Krishnamurthy et al.^[^ ^70^ ^]^ note the prominence of doping at grain surfaces and boundaries, especially with molecules, and the effect of crystallite quality (residual strain), as well as O_2_ p‐doping. Amerling et al.^[^ ^71^ ^]^ consider “surface segregation” of dopants as a problem, although molecular doping is found to be effective at grain surfaces and boundaries. They note that Bi^3+^ in MAPbBr_3_ yields rather small carrier density changes even at concentrations considered very high for classical semiconductors (1–3 ·10^3^ ppm Bi/Pb), with the *caveat* of poor reproducibility of evidence for dopant incorporation in the interior of the grains. They also note that such incorporation appears easier in nanocrystals (NC) than in bulk material. Focusing on molecular dopants, Zhang et al.^[^ ^72^ ^]^ conclude that such dopants act at surfaces and interfaces, but without apparent incorporation in the bulk material. Lin et al.^[^ ^73^ ^]^ consider the effect of the substrate / contact work function on the Fermi level position in the HaP gap, which they term “remote doping” (cf. also ref. [14], Zohar et al.), or “spatial separation of Fermi level offset from dopants”. Li et al.^[^ ^71^ ^]^ stress surface doping as the most common one for HaPs, also noting the substrate effect on doping. In a recent review devoted to doping HaPs for thermoelectrics, Chen et al.^[^ ^74^ ^]^ distinguish surface from bulk doping, where the latter includes a tabulation of bulk doping for Pb‐HaPs cases of dopant penetration via GBs into polycrystalline films. O_2_ doping on grain surfaces is discussed as well. Another recent short review by He et al.^[^ ^75^ ^]^ notes surface adsorption doping as one option among others, as well as the effect of inorganic cations in the precursor solution or deposited on the film surface during growth, on crystallite morphology. These considerations echo similar conclusions by Tabassum et al.^[^ ^76^ ^]^ and Krishnamurthy et al.,^[^ ^70^ ^]^ which stress the effect of residual (likely micro‐) strain reported, for example, for Al^3+^ addition by Wang et al.^[^ ^77^ ^]^ An earlier review of Jin et al.,^[^ ^78^ ^]^ draws attention to the dominance of surface defects, and the lack of experimental evidence for bulk defects, the occurrence of which primarily relies on computations.

### 5.2 Experimental Signatures for (Heavy) “Doping”‐Induced Bulk Structural Changes in HaPs

So, can all doping efforts for HaPs be described in terms of surface and interface effects? Not really, even though getting clear evidence for bulk incorporation of dopants is not trivial for any semiconductor, and the more so for HaPs as they are often sensitive to means used for sample preparation and/or actual characterization. Methods such as atom probe tomography (APT), ToF‐SIMS and many electron microscopy techniques, are problematic. One needs to establish maximum doses for probing particles (electron, photons, ions, etc.) below which the material is not appreciably damaged to collect relevant data. Radioactive isotopes, which might provide evidence of dopant inclusion at concentrations that are typical for classical semiconductors have been used sparingly in HaP research, and, as far as we know, not at all for this purpose. In the following we will start with results obtained for high doping levels that show evidence for bulk lattice parameter changes, which are assumed to result from dopant incorporation in the bulk of the material.

#### 5.2.1 Nanocrystals

In work on nanocrystals (NC), surface and bulk are more difficult to separate. As in the following sections on thin films and crystals, the “dopant” concentrations that are used are orders of magnitude higher than what conventionally is called doping, for semiconductors. Thus, in essence the experiments are more alike to partial substitution in the full at.% range (i.e., 10^3^–10^4^ times the ppm levels of doping in conventional semiconductors). XRD measurements on Zn:CsPbI_3_ with a Zn/Pb ratio of 1.5^[^ ^79^ ^]^ show a decrease in lattice parameter (and an increase in PLQY at 10^5^ ppm level). XRD and TEM results on CsPbCl_3_ NCs, “doped” at 7–9 at.% levels with a series of trivalent lanthanides, including Ce, Sm, Eu and Yb, for improving their optical properties, show a ≈1–2% decrease in lattice parameter.^[^ ^80^ ^]^ More recent work^[^ ^81^ ^]^ used TEM on NCs and APT on a single crystal thinned via focused ion beam (FIB) to characterize the structural effect of Yb(III) doping of CsPbCl_3_. With ≈5 at.% Yb(III), XRD showed a ≈1% decrease in lattice parameter. STEM‐EDS and APT data were interpreted as evidence for doping inside the NCs, proposing Yb to be interstitial as well as substitutional (for Pb). While the issue of avoiding beam damage is noted, no data were given for this relatively stable HaP.

#### 5.2.2 Thin Films

For polycrystalline films with larger grain sizes (few 100 nm), clear XRD evidence was found for CsPbI_2_Br lattice shrinking by ≈0.2% (≈1 pm) upon doping with 1 at.%/Pb of Zn(II) or Sb(III).^[^ ^65^ ^]^ In the same Sb(III):CsPbI_2_Br system, phase stabilization was seen, as well as evidence for a decrease in surface defect density at the 1 at.% Sb/Pb level, but the XRD results, interpreted as a decrease in lattice parameter, were marginal.^[^ ^82^ ^]^ The evidence for bulk dopant incorporation in the study of 0.7–3.5 at.%/Pb Sm(II) doping of MAPbI_3_ (MAPI) was a possible 0.5 pm increase in lattice parameter for all Sm levels, suggesting a Sm(II)→ Sm(III) oxidation, as the latter is stable in octahedral environment and, thus, can substitute for Pb(II).^[^ ^83^ ^]^ XPS showed that indeed the near‐surface contains Sm(III). The carrier density increased by 2.5–3 orders of magnitude. With Nd(III) (5 at.%/Pb) doping of FAPbI_3_, a ≈1.4% change in lattice parameter was found, with similar results for doping with Ca(II) and Na(I).^[^ ^84^ ^]^ As these changes show an increase, rather than a decrease in lattice parameter, while the effective ionic (Shannon) radii of sixfold coordinated Nd(III), Ca(II) and Na(I) are more than 15% smaller than that of Pb(II), it was assumed that all dopants occupy interstitial positions. While Nd doping at 0.8 at.%/Pb gave a 10x increase in PLQY, no data on electronic doping were given. In similar experiments,^[^ ^85^ ^]^ while no evidence was given for dopant (Nd, Ca or Na) incorporation into the grains, the stability of cells made with these ions added to the preparation solution was found to improve in the order Nd(III) > Ca(II) > Na(I).

#### 5.2.3 Single Crystals

In further examples of dopant incorporation in the bulk, doping *single crystals* of MAPbBr_3_ by 1 at.%/Pb of Bi(III) was found to yield a 1000‐fold increase in (n‐type) conductivity, with a ≈0.01% lattice parameter decrease and some increase in diffraction peak widths.^[^ ^86^ ^]^ Ag or Au doping,^[^ ^87^ ^]^ which can be justified from the existence of mixed valence noble metal halide perovskitoids^[^ ^88^, ^89^ ^]^ such as the recently reported Cs_2_Au(I)Au(III)Br_6_, was understood as electrochemical doping (in polycrystalline, i.e., grain surface‐rich, films). This is an important example because of the possible occurrence of direct Au‐ or Ag‐HaP contacts in device structures. “Near‐interface” electrochemical reactions were mentioned, while bulk doping of the grain interiors was also invoked, based on ToF‐SIMS depth profiles of the polycrystalline films. No change in lattice parameters was reported.

#### 5.2.4 Bi “Doping”

There have been several reports on doping efforts with Bi to substitute for Pb. As noted in the above‐mentioned reviews, the results are mostly inconclusive, except when orders of magnitude higher doping levels than for typical semiconductors are used (at. %, i.e., 10,000 ppm, i.e., ≈10^20^ cm^−3^). Here we note work that shows that adding Bi to the precursor solution improves the thermoelectric properties of MAPI films, using 1–5 at.% Bi.^[^ ^90^ ^]^ The absence of changes in lattice parameters was attributed to “defect tolerance”. Films made with Bi had smaller grain size and a slightly increased temperature for the tetragonal to cubic phase transition than films made without Bi. Most changes that could be observed were at the grain surfaces and boundaries. From the frequency dependence of the film capacitance, it was suggested that differences between bulk and surface could be separated.

### 5.3 Organic Molecular “Doping

As mentioned earlier, organic molecules present options for surface doping, as reviewed by Zhang et al.^[^ ^72^ ^]^ In further recent work,^[^ ^91^ ^]^ the *molecular charge transfer* effect was studied and p‐doping of MA(Pb_0.5_Sn_0.5_)I_3_ was found with F_4_TCNQ located mostly at GBs and surfaces. In other work,^[^ ^92^ ^]^ 3‐(aminomethyl)pyridine was reacted with (nominally) Rb_0.05_MA_0.05_FA_0.85_Pb[I_0.95_Br_0.05_]_3_ and a smaller work function, meaning a more n‐type material resulted, which was explained by (surface) doping. The treatment also made the films smoother, which was proposed to lead to a shallower surface potential landscape than for rougher surfaces. From static DFT computations, the V_I_ enthalpy of formation in such landscape was found to be decreased,^[^ ^92^ ^]^ compared to that in the bulk, which fit with observed surface halide deficiency. In a further conjecture, these results were taken to explain the improved PV performance of cells made with this surface reagent. These results, especially those concerning HaP surface modifications to get very high PLQYs with polycrystalline films of MAPbI_3_ with mild chemical treatments^[^ ^93^ ^]^ or nanoparticles of HaPs,^[^ ^94^, ^95^ ^]^ as well as the ability to use molecular dopants on surfaces^[^ ^96^, ^97^ ^]^ or at grain boundaries^[^ ^91^ ^]^ to affect the charge density in the bulk, fit with the premise that *volume doping of HaPs is possible by way of controlled surface doping*.

### 5.4 Fermi Level Pinning and Doping

We recall that E_F_ pinning can result from aggressive defect‐inducing treatments, such as direct metal or metal oxide evaporation^[^ ^98^, ^99^ ^]^ onto a semiconductor surface, while E_F_ can move through much of the band gap of various HaPs by changing the work function of the substrate onto which the HaP is deposited. Schulz et al.^[^ ^19^ ^]^ and Endres et al.^[^ ^17^ ^]^ demonstrated 0.7–1.0 eV E_F_ shifts in MAPbI_3_ and CsPbBr_3_, respectively, when films of these compounds are spin‐coated on TiO_2_ (low work function) vs. NiO_x_ (large work function). Zohar et al.^[^ ^14^ ^]^ (Figure 1) and Shin et al.^[^ ^18^ ^]^ pointed out the linear dependence of the work functions of (FA, MA)PbBr_3_ and MAPbI_3_, respectively, on the work function of a wide range of substrate contacts. Noel et al.^[^ ^20^ ^]^ showed a one‐to‐one correspondence over 0.7 eV between the work function of FA_0.83_MA_0.17_Pb(I_0.83_Br_0.17_)_3_ films and that of the SnO_2_ substrate treated with increasing amounts of tetrafluoroborate‐based ionic liquid. As already alluded to in the introduction, these experiments unambiguously demonstrate critical aspects of the surfaces, interfaces and bulk properties of HaPs:

i) the interfaces and bulk of these HaP films have very low densities of deep E_F_ pinning states;
ii) HaP films have low to very low (mostly < 10^15^ cm^−3^) intrinsic carrier densities. Charge exchange between substrate and films to establish thermal equilibrium can shift the HaP Fermi level throughout the few 100 nm of the film thickness. To get a sense for the maximum carrier densities generated in these materials when E_F_ spans from 0.3 eV below the CB edge to 0.3 eV above the VB edge, we can assume typical effective densities of states at the CB or VB edges of *N* = 10^18^ cm^−3^. Then, the electron density, $n=Nexp(-\frac{E_{C}-E_{F}}{kT})$, or hole density, $p=Nexp(-\frac{E_{F}-E_{V}}{kT})$, remains < 10^13^ cm^−3^.

Other key experiments demonstrating the link between surface defects and E_F_ pinning were done on MAPbI_3_ illuminated with white light in vacuum (cf. explanatory note ‐D‐) and showing the formation of Pb^0^ and E_F_ pinning close to the conduction band edge.^[^ ^100^ ^]^ The subsequent “passivation” of the defects using the oxidizing molecule F_4_‐TCNQ to re‐oxidize the under‐coordinated Pb was shown to unpin E_F_ and eliminate much of the surface band bending.

### 5.5 Effects Due to Oxygen and Water

Finally, there is the effect of O_2_ and H_2_O exposure of HaP films and crystals, which was already mentioned in ‐4‐ above for other semiconductors. Oxygen (O_2_) is found to be an effective p‐type dopant^[^ ^18^, ^101^, ^102^ ^]^ and the doping is reversible, i.e., the density of holes changes with the partial pressure of O_2_ in the ambient to which the sample is exposed. Furthermore, O_2_ can change the density and even dominant type of electronic carriers in HaPs. Several reports consider the effect of O_2_, and of O_2_ with H_2_O, on the PL of single crystals and polycrystalline thin films,^[^ ^103^ ^]^ showing the PL in air to increase, with the strongest effect from O_2_ and that the effect extends to electronic conduction.^[^ ^104^ ^]^ The latter was shown by Halder et al. to be concomitant with a change in Seebeck coefficients.^[^ ^102^ ^]^ This was explained through defect interactions via physisorption, in analogy to what was found for other semiconductors, explaining readily the reversibility of the exposure process. Importantly, decreasing crystallite size was also found to increase the effect if polycrystalline films were used,^[^ ^102^, ^104^ ^]^ The O_2_‐adsorption effect exists also for single crystals measured between two lateral contacts.

**Figure 5:**
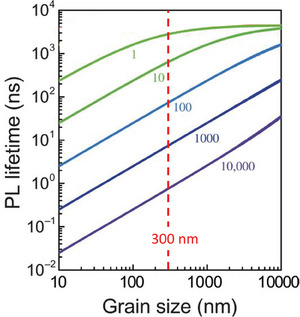
Photoluminescence, PL, lifetimes (from time‐resolved PL decay) in polycrystalline thin films or small crystals for surface recombination velocities (SRV) from 1 to 10^4^ cm/s, taken from ref. 104]. The carrier diffusion coefficient D is taken as 5.3 cm^2^ sec^−1^ and a bulk carrier lifetime τ_b_ = 4.5 µs, the value approached at the top right for SRV 1–10 cm sec^−1^ and macroscopic single crystal sizes. As noted in ref. [104], the surface state density would then be < 1 ppm (cf. Figure 2 above). Note that the lowest SRV found for any semiconductor, for fully passivated c‐Si, is 0.25 cm sec^−1^ For halide perovskites (vertical red line was added to show typical grain size in HaP thin films for PV), values as low as 10 cm s^−1^ have been reported. These calculations thus imply a strong grain size effect on the electronic characteristics of HaP grains up to at least several µm. Adapted from Science Advances ref. [104]. The Authors, some rights reserved; exclusive licensee AAAS. Distributed under a CC BY‐NC 4.0 license http://creativecommons.org/licenses/by‐nc/4.0/. Reprinted with permission from AAAS.

For single crystals, using 2‐photon spectroscopy, reversible O_2_‐induced p‐doping was also shown to be present, although somewhat decreased, in the bulk of the samples.^[^ ^104^ ^]^ As shown in **Figure** **5** (ref. Fang et al.^[^ ^104^ ^]^) the observed effect can be explained in terms of a change in surface recombination velocity.^[^ ^105^ ^]^ While it is possible that O_2_ or other gases also interact with bulk native defects to actually volume‐dope the material,^[^ ^18^, ^106^ ^]^ the rapid and facile response to changes in atmosphere implies a significant, likely dominant surface component. Also, in contrast to the case for H_2_O,^[^ ^107^ ^]^ there is as yet no experimental evidence for the presence of O/O_2_ in the bulk of HaPs.

## 6 Summary and Conclusion

We presented the arguments and experimental data for the case that, at least to a first approximation, volume electrical properties of Pb‐HaP films are controlled by way of their surfaces. Using literature data, we noted that this phenomenon is much more pronounced for Pb‐HaPs than for other semiconductors, such as CIGS and other Cu‐chalcopyrites, and in these HaPs it will normally dominate.

Sn‐HaPs and Sn/Pb‐HaPs were not included in our analysis because in these uncontrolled n‐doping, often over‐doping, due to Sn(II) oxidation is still the rule.^[^ ^108^ ^]^ With efforts focused on eliminating this unwanted doping, the basic conditions for dominant surface doping, i.e., low bulk and surface densities of defects as dopants, have not yet been met.

In double Ag‐Bi HaPs, few reports focused on dopant compensation or modifying the bandgap for specific applications, such as detectors, but also here the above‐mentioned conditions are not met.^[^ ^109^, ^110^ ^]^ Therefore, at this stage of research, little to no information is available regarding the control of doping via the surface for these other Sn‐containing HaP compounds or double halide perovskites.

The key enabler in the Pb‐HaPs is the intrinsically low free bulk carrier density due to low doping levels, which, in turn, result from strong lattice dynamics that still allow high crystalline order on *average*. These dynamics also lie at the core of the self‐healing and defect tolerance in HaPs. The low doping levels lead to space charge layer widths larger than the diameter and thickness of the average grains in polycrystalline thin films, which keep the grains depleted. While examples of change in bulk material (grain interior in case of polycrystalline films) are observed upon dopant incorporation, these few cases, evidenced by a change in carrier density derived from resistivity measurements accompanied by a change in lattice parameters, require dopant concentrations at the at. % (per Pb for Pb‐HaPs). These are concentrations many orders of magnitude higher than common semiconductor doping levels. The low doping levels in Pb‐HaPs imply low defect densities, both in the bulk and on the surfaces. The latter is critical for understanding the widely reported strong effects of chemical surface passivation on photoluminescence and, as the passivated surface is transformed into a junction interface, for device performance. Only if low bulk defect concentrations are accompanied by low surface defect concentrations can the dramatic passivation effects be rationalized and understood. We conclude that any result relevant to HaP doping should first be examined as possible surface effects. Only after those are excluded can bulk doping be considered. The same logic applies to assumed incorporation of certain isovalent substitutions into HaPs (e.g., Rb, pseudo‐halides).

## Explanatory Notes

### **‐A‐** Defect Density and Depletion Width

Estimated number of defects in a depletion region, *W*, of 100 nm (Equation (1) in main text):

(A1) $$\begin{array}{c} N_{D}=\frac{2\varepsilon_{s}V}{qW^{2}}=\frac{3.3\cdot 10^{8}}{W^{2}}\left[m^{-3}\right]\approx 3\cdot 10^{22}\left[m^{-3}\right]=3\cdot 10^{16}\left[cm^{-3}\right] \end{array}$$

where we took, in this rough calculation, *V* = 0.1 V and ɛ_*s*_ ≈ 30 ɛ_*o*_, with the permittivity in vacuum ɛ_*o*_ ≈ 8.9 · 10^−12^ F/m (= C/V·m) and the electron charge q ≈ 1.6 · 10^−19^ C.

### **‐B‐** Surface and Volume Defect Densities

Calculation of how surface defect density translates into a volume density for a sphere of radius *r*:^7^

*Definitions*: *n_surf_* · 4π ·  *r* ^2^ = *N_surf_* ; r [cm]; *n_surf_* [cm^−2^]; *N_surf_ is the total number of defects on the surface*

4π · *a* ^2^ · 10^−8^ ·   *n*_surf_ = *N*_surf_; a [µm], (i.e., the value of r in [µm], instead of [cm])

(B1) $$n_{\mathrm{surf}}=N_{\mathrm{surf}}/4\pi \cdot a^{2}\cdot 10^{-8}$$

$n_{vol}=4\pi \cdot a^{2}\cdot 10^{-8}\cdot \;n_{\mathrm{surf}}/\frac{4}{3}\pi \cdot a^{3}\cdot 10^{-12}\;$; *n_vol_* [cm^−3^] is the volume defect density, solely due to *N_surf_*.

(B2) $$n_{vol}=3\cdot 10^{4}\cdot n_{\mathrm{surf}}/a$$

*Example*:

Take *r* = 150–300 nm, i.e., d = 2*r* = 300–600 nm, i.e., a = 0.15–0.3 µm.

 T*o* get *n*_vol_ = 10^13 ^ →  10^17^ [cm^−3^]; take *n*_vol_ = 1 − 2 · 10^5^ *n_surf_*

 *n*_surf_ = 10^8^ – 10^12^ [cm^−2^], if ≤ 1 ppm – ≤ 1% of all the surface sites are electrically active.

Then, because *n_vol_* = 1 − 2 · 10^5^ *n_surf_* → *n_vol_* = 1 − 2 · 10^13^ − 10^17^ [cm^−3^]

Similar back‐of‐the‐envelope calculations or figures that express such, can be found throughout the literature, and for HaPs in, for example, refs. [34, 35].

### **‐C‐** Classical Point Defect Chemical Example of HaP Surface Doping

We use Kröger‐Vink notation: V_Z_ represents a vacancy of Z in the lattice; Z_i_ is Z on an interstitial lattice site; and X_Z_ means X on a Z lattice site. Except for electrons and holes in the bands (subscripts _CB_, _VB_), charges are with respect to the neutral lattice, and indicated by superscripts *^x^*, ′ and ^•^, for neutral, singly negative and singly positive, respectively. Following ref. [50] for CIGS chalcopyrites, the process can be written as:

(C1) $$\begin{array}{c} \text{…}\text{…}\left\{{\mathrm{Pb}}_{\mathrm{Pb}}^{x}-I_{I}^{x}\right\}<===>\cdots \cdots \left\{{\mathrm{Pb}}_{\mathrm{Pb}}^{x}-V_{I}^{x}\right\}+\left[\frac{1}{2}I_{2}\right]\\ bulk\;surf\;\;\;\;\;\;\;bulk\;\;surf\;\;\;gas \end{array}$$

(C2) $$\begin{array}{c} \text{…}\text{…}\left\{P{b_{\mathrm{Pb}}}^{x}-V_{I}^{x}\right\}<===>+\;e_{\mathrm{CB}}^{-}+\cdots \cdots \left\{{\mathrm{Pb}}_{\mathrm{Pb}}^{x}-V_{I}^{•}\right\}\\ bulk\;surf\;\;\;\;\;\;\;\;\;\;\;\;bulk\;\;surf \end{array}$$

(Partial) reaction 1a gives a schematic idea of a halide vacancy creation upon surface formation. (Partial) reaction 1b shows how a positive surface charge (superscript •) is formed as a result of charging of the I surface vacancy ($V_{I}^{\;x}$ in Equation (C1)), created upon surface formation: $V_{I}^{\;x}$≤ = > $V_{I}^{\cdot }$ + $e_{\mathrm{CB}}^{-}$. Physical electronic properties of films, measured before exposure to an oxidizing species, should thus reflect grains and their surfaces in this condition; 1a + 1b comprise an oxidation/reduction process (Equation (C3)):

(C3) $$\begin{array}{c} \text{…}\text{…}\left\{{\mathrm{Pb}}_{\mathrm{Pb}}^{x}-I_{I}^{x}\right\}<===>e_{\mathrm{cb}}^{-}+\cdots \cdots \left\{{\mathrm{Pb}}_{\mathrm{Pb}}^{x}-V_{I}^{•}\right\}+\left[\frac{1}{2}I_{2}\right]\\ bulk\;surf\;\;\;\;\;\;\;\;\;bulk\;\;surf\;\;\;\;gas \end{array}$$

as a result of which the bulk is reduced and the surface is oxidized.

The oxidized surface will be positively charged and, if band bending is possible, it will be downward toward the surface (p‐type), in the near‐surface. In HaP grains, given their small size and the bulk doping densities, no significant band bending is normally possible.

Chlorination (or similar processes, incl. oxygenation) can neutralize positively charged sites and can be written as:

(C4) $$\begin{array}{c} \text{…}\text{…}{\left\{{\mathrm{Pb}}_{\mathrm{PB}}^{x}-V_{I}^{•}\right\}}_{\mathrm{surf}}{\left\{Cl^{-}\right\}}_{\mathrm{abs}}<===>\cdots \cdots \left\{{\mathrm{Pb}}_{\mathrm{Pb}}^{x}-{\mathrm{Cl}}_{I}^{x}\right\}\\ bulk\;\;\;surf\;\;\;\;\;\;\;\;\;\;bulk\;\;surf \end{array}$$

where the subscript “_ads_” means an adsorbed species. As a result of (3) the bulk is now oxidized and the surface is reduced. This is readily seen by including the Chlorine reduction, needed to form Cl^−^:

(C5) $$\frac{1}{2}\;Cl_{2}+{\;e}_{\mathrm{CB}}^{-}\leq =>{\left\{Cl^{-}\right\}}_{\mathrm{abs}}$$

Note that, due to the significant electronegativity difference between chloride or iodide and Pb(II) in the dihalides,^[^ ^111^ ^]^ ≈0.5–0.6 on the Pauling scale, the product of process (3) should really be written as

(C6) $$\cdots \cdots \cdots {\left\{{\mathrm{Pb}}_{\mathrm{Pb}}^{\;\partial +}-{\;\mathrm{Cl}}_{I}^{\;\partial -}\right\}}_{{\mathrm{surf}}^{•}}$$

Reaction (3) leads to oxidation of the bulk (by taking delocalized electrons from the CB (cf. Equation (C5)) and reduction of the surface (by localizing electrons on the Cl, on the surface). This should reduce the n‐ and increase p‐type character of the HaP.

### **‐D‐** Fermi Level Pinning

Given radiation of AM 1.5G, the solar flux absorbed by a 1.5 eV bandgap material is ≈$10^{17}\frac{\;\#\;photons}{cm^{2}\cdot sec}$ (*J_sc_^max^* = 29 mA/cm^2^, using ASTM G173‐03).^[^ ^112^ ^]^

Assuming (for simplicity) that:

– all these photons are absorbed within 0.5 µ*m*
– the average carrier lifetime is 1 µ*sec*

then the steady‐state carrier density will be ≈10^16^ *cm* ^−3^. Therefore, if the defect density is lower than this value, we will not observe a pinning.

## Conflict of Interest

The authors declare no conflict of interest.
